# Testing flexible polymer solar cells in near-space

**DOI:** 10.1093/nsr/nwad071

**Published:** 2023-03-14

**Authors:** Peter Müller-Buschbaum

**Affiliations:** Department of Physics, TUM School of Natural Sciences, and Heinz Maier-Leibnitz-Zentrum (MLZ), Technical University of Munich, Germany

Since the big success of the Vanguard 1 mission, being the first solar-powered satellite, the use of solar cells in space developed very rapidly. Today, conventional solar cell technologies for space are based on silicon and GaAs modules. If significantly higher power conversion efficiencies (PCEs) are required, sophisticated multiple junction devices are in use such as III–V triple-junction GaInP/GaAs/GaInAs modules. However, such inorganic solar cells are expensive and supported on rigid substrates, not flexible and rather heavy, also considering the massive deployment systems typically needed. Accordingly, the specific power or power-per-weight, i.e. the ratio of generated power per mass (measured in W kg^−1^), is very limited despite high PCE values [1].

Due to high launch costs and payload weight limits, a strong increase in the specific power of solar cells for space use is highly desirable. Moreover, truly flexible solar cells will enable completely different deployment strategies. Novel solar cell technologies based on organic solar cells or perovskite solar cells offer these advantages of being lightweight, potentially cheap and fully flexible [2]. Together with a wide range of tunability, these novel technologies become highly attractive for space use, after terrestrial tests demonstrated a very promising stability in laboratory space simulations (testing vacuum & AM0/radiation tolerance) [3,4]. First conceptual tests were realized with balloon [5,6] and rocket flights [7], which explored 35- and 240-km altitudes, respectively.

In particular, the near-space region has gained high interest for high-altitude pseudo satellites or near-space aircrafts in recent years and organic solar cells or perovskite solar cells appear highly promising to power objects in the stratosphere. Recently, a team led by Chang-Qi Ma, Zhixiang Wei, Qun Luo and Guoning Xu published excellent and solid work on *in situ* performance and stability tests of large-area flexible polymer solar cells in the 35-km stratospheric environment [8]. It is to highlight that fully flexible organic solar cells (for device geometry, see Fig. 1a) were studied *in situ* with respect to performance and stability at 35-km altitude with a high-altitude balloon measurement system (Fig. 1b). These balloon flight data were compared with systematic terrestrial investigations on the performance under thermal cycling and intense UV irradiation. Before and after flying in near-space as well as under terrestrial conditions, the PCEs were compared (Fig. 1c) and the authors concluded that flexible organic solar cells can resist the extreme environment of near-space. Of special value are the rich solar cell device data collected during the flight, probing different conditions in terms of temperature and irradiation intensity as seen in Fig. 1d. Here, a single balloon flight is capable of probing different conditions, thereby being an excellent test platform.

**Figure 1.**
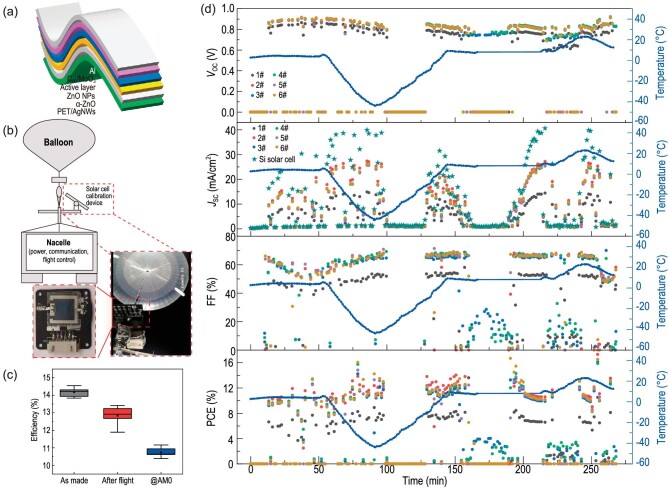
(a) Sketch of inverted device structure of AgNWs/α-ZnO/ZnO NPs/PBDB-T-2F : BTP-4F/C60/MoO_3_/Al with an area of 0.64 cm^2^ fabricated on 125- or 38-μm thin flexible PET substrates; (b) schematic diagram of the high-altitude balloon measurement system including photographs of the balloon and device test system; (c) efficiency before and after balloon flight and at AM0 for six solar cells; (d) temporal evolution of open-circuit voltage *V*_OC_, short-circuit current *J*_SC_, fill factor FF and PCE during the balloon flight. Reprinted from Ref. [8].

In summary, this timely research article demonstrates the power of using near-space balloon studies for probing next-generation solar cells and highlights the large potential of flexible organic solar cells for use in the stratosphere.

## Conflict of interest statement

None declared.
